# Multi-Targeted Approaches and Drug Repurposing Reveal Possible SARS-CoV-2 Inhibitors

**DOI:** 10.3390/vaccines10010024

**Published:** 2021-12-26

**Authors:** Khalid Mashay Alanazi, Mohammad Abul Farah, Yan-Yan Hor

**Affiliations:** 1 Department of Zoology, College of Science, King Saud University, Riyadh 11451, Saudi Arabia; kalanzi@ksu.edu.sa (K.M.A.); mfarah@ksu.edu.sa (M.A.F.); 2 Department of Biotechnology, Yeungnam University, 280 Daehak-ro, Gyeongsan 38541, Gyeongbuk-do, Korea

**Keywords:** COVID-19, drug repurposing, multi-targeted inhibitors, structural proteins, non-structural proteins

## Abstract

The COVID-19 pandemic caused by SARS-CoV-2 is unprecedented in recent memory owing to the non-stop escalation in number of infections and deaths in almost every country of the world. The lack of treatment options further worsens the scenario, thereby necessitating the exploration of already existing US FDA-approved drugs for their effectiveness against COVID-19. In the present study, we have performed virtual screening of nutraceuticals available from DrugBank against 14 SARS-CoV-2 proteins. Molecular docking identified several inhibitors, two of which, rutin and NADH, displayed strong binding affinities and inhibitory potential against SARS-CoV-2 proteins. Further normal model-based simulations were performed to gain insights into the conformational transitions in proteins induced by the drugs. The computational analysis in the present study paves the way for experimental validation and development of multi-target guided inhibitors to fight COVID-19.

## 1. Introduction

Starting from one patient in December 2019 at Wuhan city of China, COVID-19 has caused mayhem worldwide. As of 24 April 2020, SARS-CoV-2, the etiological agent of COVID-19 has infected 165,069,258 people causing 3,422,907 deaths globally (as of 21 May 2021) [1]. The situation is further worsened by variants of COVID-19 circulating in the global population, which have tremendously increased the transmission rate of the virus [2]. Apart from mutational, structural, and phylogenetic analyses of the SARS-CoV-2 genome, scientists have been centering on drug repurposing to develop therapeutics to combat SARS-CoV-2 contagion [3]. Various existing drugs, Remdesivir, Lopinavir/Ritonavir, Interferon beta-1a, Chloroquine/hydroxychloroquine, are under SOLIDARITY trial initiated by WHO for their inhibitory activity against different proteins of SARS-CoV-2 [4,5] nevertheless better targeted inhibitors are required for COVID-19 treatment.

SARS-CoV-2 genome is known to encode up to 14 open reading frames that translate to structural proteins, spike (S), membrane (M), envelope (E) and nucleocapsid (N); two huge non-structural proteins (NSPs) cleaving into sixteen smaller proteins along with nine accessory factors. The virus uses S protein to bind to the angiotensin-converting enzyme 2 (ACE2) receptor to enter the host cell. Both the M and E proteins are involved in forming the virus envelope and the pathogenesis of the virus, while the N protein binds to the virus’s RNA genome, creating the nucleocapsid [6]. NSPs form the replication/transcription complex that includes the papain-like proteinase (NSP3), the main proteinase (NSP5), the NSP7-NSP8 complex, the RNA-dependent RNA polymerase (NSP12), a NTPase/helicase (NSP13), an exonuclease (NSP14), an endonuclease (NSP15), and 2′O-methyltransferases (NSP16). Another group of SARS-CoV-2 is the accessory proteins, 3a, 3b, 6, 7a, 7b, 8, 9b, 9c and 10. The accessory proteins serve multitude of functions in virus replication [7]. In earlier studies, several SARS-CoV-2 proteins have been identified as having high mutational propensity, which further demands for multi-targeted inhibitors [8].

In the present study, virtual screening followed by normal model-based simulations was performed using nutraceuticals against 14 SARS-CoV-2 proteins; NSP1, NSP3, NSP5, NSP9, NSP12, NSP13, NSP15, 3a, S, E, M, 6, 7a and N that led to the identification of compounds that can be repurposed against SARS-CoV-2. The interaction analyses further exhibited the hydrogen bonding and hydrophobic network forming residues between protein and ligand. The findings of the proposed work will aid in global efforts for fight this pandemic by expediting drug development against COVID-19.

## 2. Methodology

### 2.1. Protein Structure Modelling and Preparation

The X-ray structures of some SARS-CoV-2 proteins, N, NSP3, NSP5, NSP9 and NSP15, are available on protein data bank (PDB) [9] corresponding to PDB ids 5MM3, 6W9C, 5RED, 6W4B and 6VWW, respectively. Homology modelling was performed for the remaining proteins using SWISS-MODEL server which identifies the structural templates followed by the alignment of query protein sequence with template structures and model generation. Prior to docking studies, the proteins were processed using Schrodinger Protein Preparation Wizard during which missing hydrogens were added, hydrogen bonds were optimized, and water molecules were deleted [10].

### 2.2. Nutraceuticals (Ligands) Preparation

Nutraceuticals are bioactive phytochemicals that deliver health benefits and are relatively safe for prevention and treatment against the disease. Nutraceuticals have already been used for treating several diseases, including atherosclerosis, cancer, cardiovascular diseases, diabetes, hypertension, inflammation, obesity, and others. A large number of nutraceuticals are common with FDA-approved pharmaceutical drugs. The library of 150 nutraceuticals was obtained from the DrugBank database [11]. The chemical structures of nutraceuticals were prepared with LigPrep module Schrodinger which generated diverse, accurate, and energy minimized conformations using the OPLS-2005 force field [10].

### 2.3. Docking Studies

A rigorous virtual screening was performed using Schrodinger Glide (grid-based ligand docking with energetics) [12]. Furthermore, docking studies for the top-scoring compounds were also done using GOLD (Genetic Optimization for Ligand Docking) [13] and AutoDock Vina [14] software.

### 2.4. Docking Studies Using Glide

Glide uses a range of filters and thoroughly searches the conformational, orientational, and positional space for ligand in the receptor’s binding site. The receptor was represented by creating a cubic grid of size 30 × 30 × 30 Å centered on the active site residues for each protein by Schrodinger’s Receptor Grid Generation program [12]. Further, the ligands were screened against each receptor using Glide’s high throughput virtual screening (HTVS) approach [12]. The high-ranking ligands were then subjected to stringent screening via the extra precision (XP) [12] method to eliminate false positives and acquire distinct binding modes of compounds.

### 2.5. Docking Studies Using GOLD

GOLD employs a genetic algorithm to examine the conformational flexibility of the ligand and partial (side-chain) flexibility of the receptor and generates the accurate binding mode of ligands [13]. The binding site for the ligands was specified as a ligand-specific pocket involving all the active site residues of protein within a 6 Å radius. The docked poses of ligand were evaluated using GoldScore fitness function, which considers protein-ligand hydrogen bonding and van der Waals energies along with ligand internal and torsional strain energy.

### 2.6. Docking Studies Using AutoDock Vina

AutoDock Vina uses a gradient optimization method to find low energy docked conformations of ligand [14]. A grid box of size 70 × 70 × 70 Å along X, Y and Z axes was generated considering active site residues of the protein. The lowest binding affinity ligand pose was separated and complexed with the receptor.

### 2.7. Interaction Analyses

LigPlus was used to compute interactions between the docked protein-ligand complexes. 2D diagrams were generated depicting hydrogen bonds and hydrophobically interacting residues.

### 2.8. Normal Mode Analyses

To study the conformational variations upon ligand binding in the protein, normal mode analyses (NMA) was performed using a rigid cluster NMA based NMSim web server [15]. NMA has been used to predict high amplitude conformational transitions and correlated atomic movements induced in the protein on the binding of a ligand, which largely occurs near the lowest energy state of the unbound protein [16,17]. The protein acquires a favorably compact state on binding a ligand, measured by radius of gyration (ROG) [18]. Hence, ROG-guided simulation implemented in NMSim was applied with default parameters. The NMSim approach is performed in three steps: a rigid cluster decomposition (RCD) is obtained in the first step (FIRST analyses) followed by calculation of normal modes using rigid cluster normal-mode analysis (RCNMA). These normal modes are then used in the simulation performed by NMSim [15]. The information regarding the conformational changes of the protein during the simulation was provided by root-mean-square-deviation (RMSD) and root-mean-square-fluctuation (RMSF) graphs.

## 3. Results and Discussion

### 3.1. Molecular Docking Analyses

The compounds that showed high binding affinity against SARS-CoV-2 proteins include Rutin (NSP1, NSP3, NSP5, NSP9, NSP12, NSP13, NSP15, ORF3a, S, E, M, ORF6, ORF7a, N); NADH (NSP1, NSP3, NSP5, NSP9, NSP12, NSP13, NSP15, ORF3a, S, E, M, ORF6, ORF7a, N); Ginsenoside Rg1 (NSP1, NSP3, NSP5, NSP9, NSP12, NSP15, E, ORF5, ORF7a and N); Ginsenoside Rb1 (NSP5, NSP12, ORF7a, NSP9, NSP15); Ginsenoside C (N, ORF6 and NSP1); Spermine (ORF6); Glutathione (NSP13); Ornithine (ORF3a), and α-tocopherol succinate (E). However, the top-scoring compounds against most of the SARS-CoV-2 proteins were Rutin (DB01698), NADH (DB00157), and Ginsenoside Rg1 (DB06750) (Table 1 and Table 2).

### 3.2. Simulations Analyses

**Figure 1:**
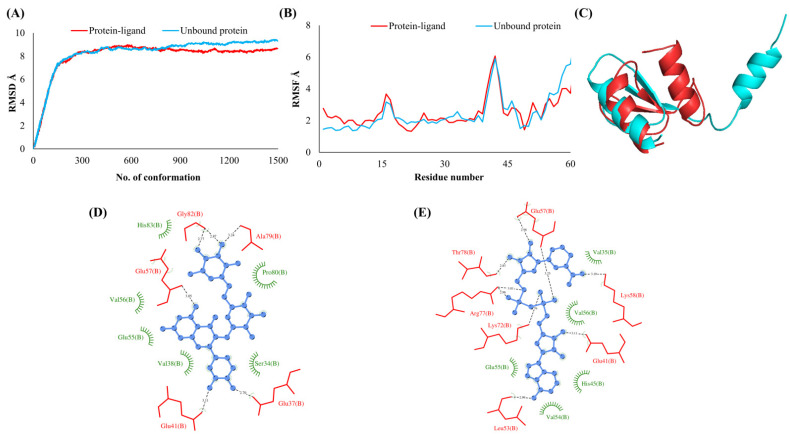
Leader protein (NSP1). (**A**) RMSD plot of unbound and ligand-bound protein (**B**) RMSF plot of unbound and ligand-bound protein (**C**) Superimposition of unbound and ligand-bound protein (**D**) Molecular interactions of protein with Rutin (**E**) Molecular interactions of protein with NADH.

**Figure 2:**
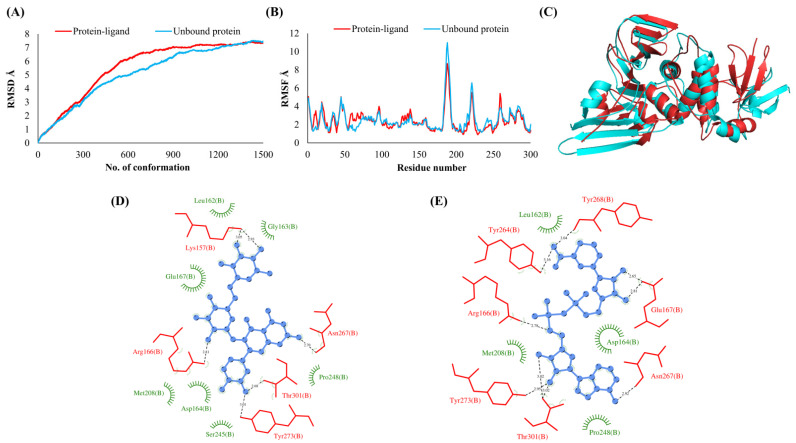
Papain-like protease (NSP3). (**A**) RMSD plot of unbound and ligand-bound protein (**B**) RMSF plot of unbound and ligand-bound protein (**C**) Superimposition of unbound and ligand-bound protein (**D**) Molecular interactions of protein with Rutin (**E**) Molecular interactions of protein with NADH.

**Figure 3:**
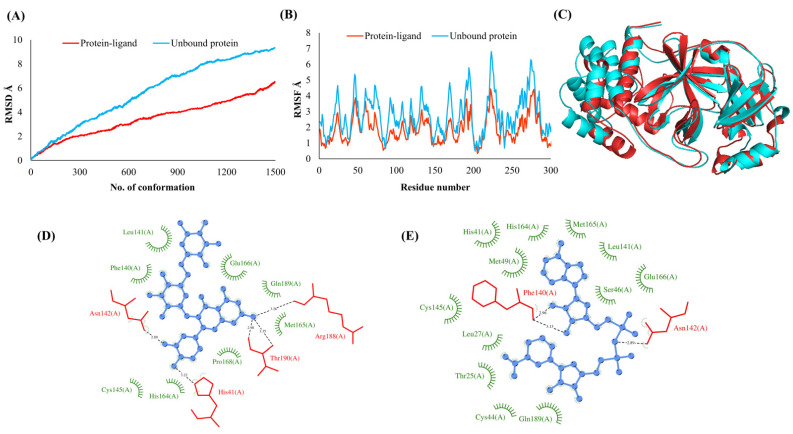
Main protease (NSP5). (**A**) RMSD plot of unbound and ligand-bound protein (**B**) RMSF plot of unbound and ligand-bound protein (**C**) Superimposition of unbound and ligand-bound protein (**D**) Molecular interactions of protein with Rutin (**E**) Molecular interactions of protein with NADH.

**Figure 4:**
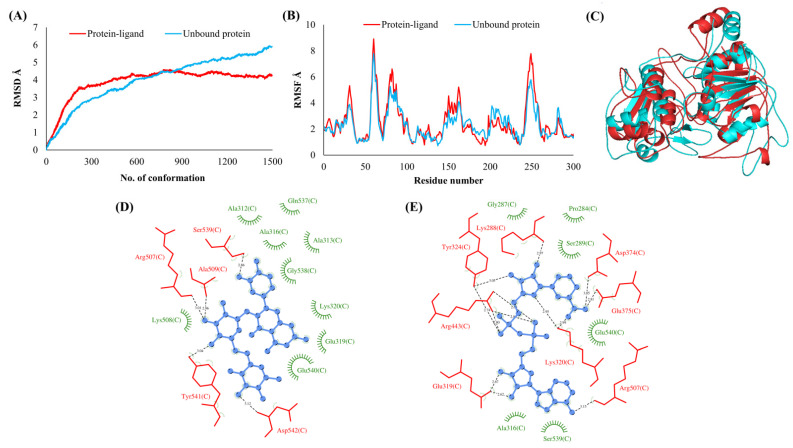
RNA binding protein (NSP9). (**A**) RMSD plot of unbound and ligand-bound protein (**B**) RMSF plot of unbound and ligand-bound protein (**C**) Superimposition of unbound and ligand-bound protein (**D**) Molecular interactions of protein with Rutin (**E**) Molecular interactions of protein with NADH.

**Figure 5:**
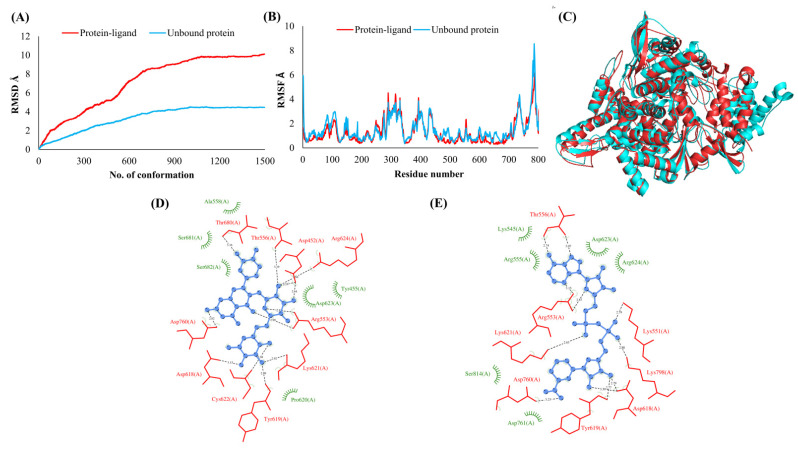
RNA-dependent RNA polymerase (NSP12). (**A**) RMSD plot of unbound and ligand-bound protein (**B**) RMSF plot of unbound and ligand-bound protein (**C**) Superimposition of unbound and ligand-bound protein (**D**) Molecular interactions of protein with Rutin (**E**) Molecular interactions of protein with NADH.

**Figure 6:**
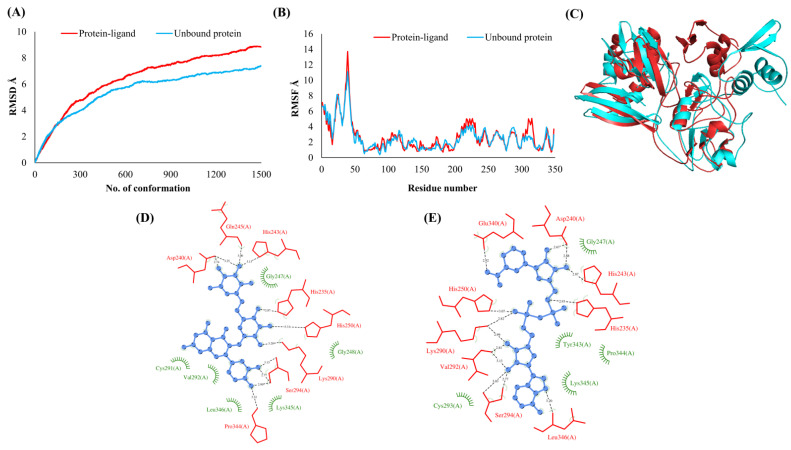
Helicase (NSP13). (**A**) RMSD plot of unbound and ligand-bound protein (**B**) RMSF plot of unbound and ligand-bound protein (**C**) Superimposition of unbound and ligand-bound protein (**D**) Molecular interactions of protein with Rutin (**E**) Molecular interactions of protein with NADH.

**Figure 7:**
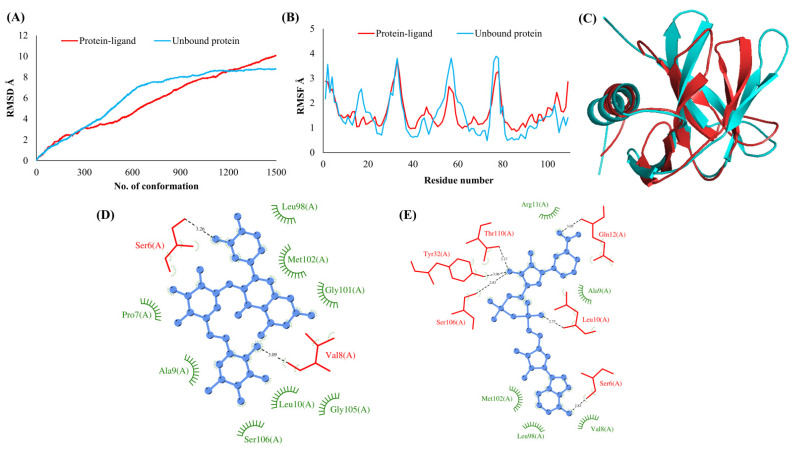
Endoribonuclease (NSP15). (**A**) RMSD plot of unbound and ligand-bound protein (**B**) RMSF plot of unbound and ligand-bound protein (**C**) Superimposition of unbound and ligand-bound protein (**D**) Molecular interactions of protein with Rutin (**E**) Molecular interactions of protein with NADH.

**Figure 8:**
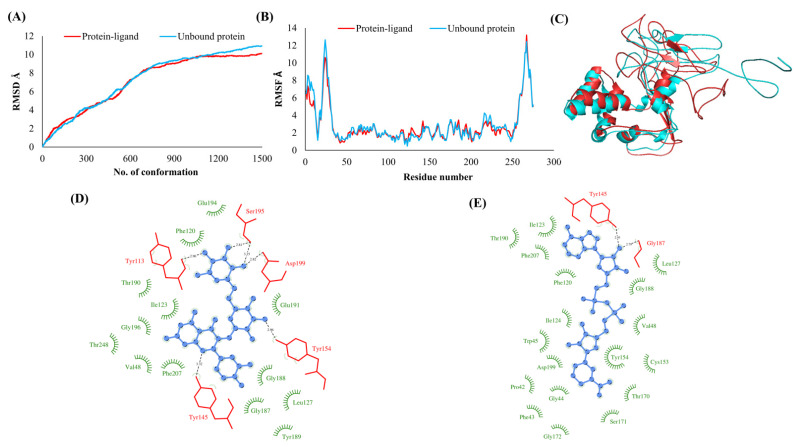
ORF 3a. (**A**) RMSD plot of unbound and ligand-bound protein (**B**) RMSF plot of unbound and ligand-bound protein (**C**) Superimposition of unbound and ligand-bound protein (**D**) Molecular interactions of protein with Rutin (**E**) Molecular interactions of protein with NADH.

**Figure 9:**
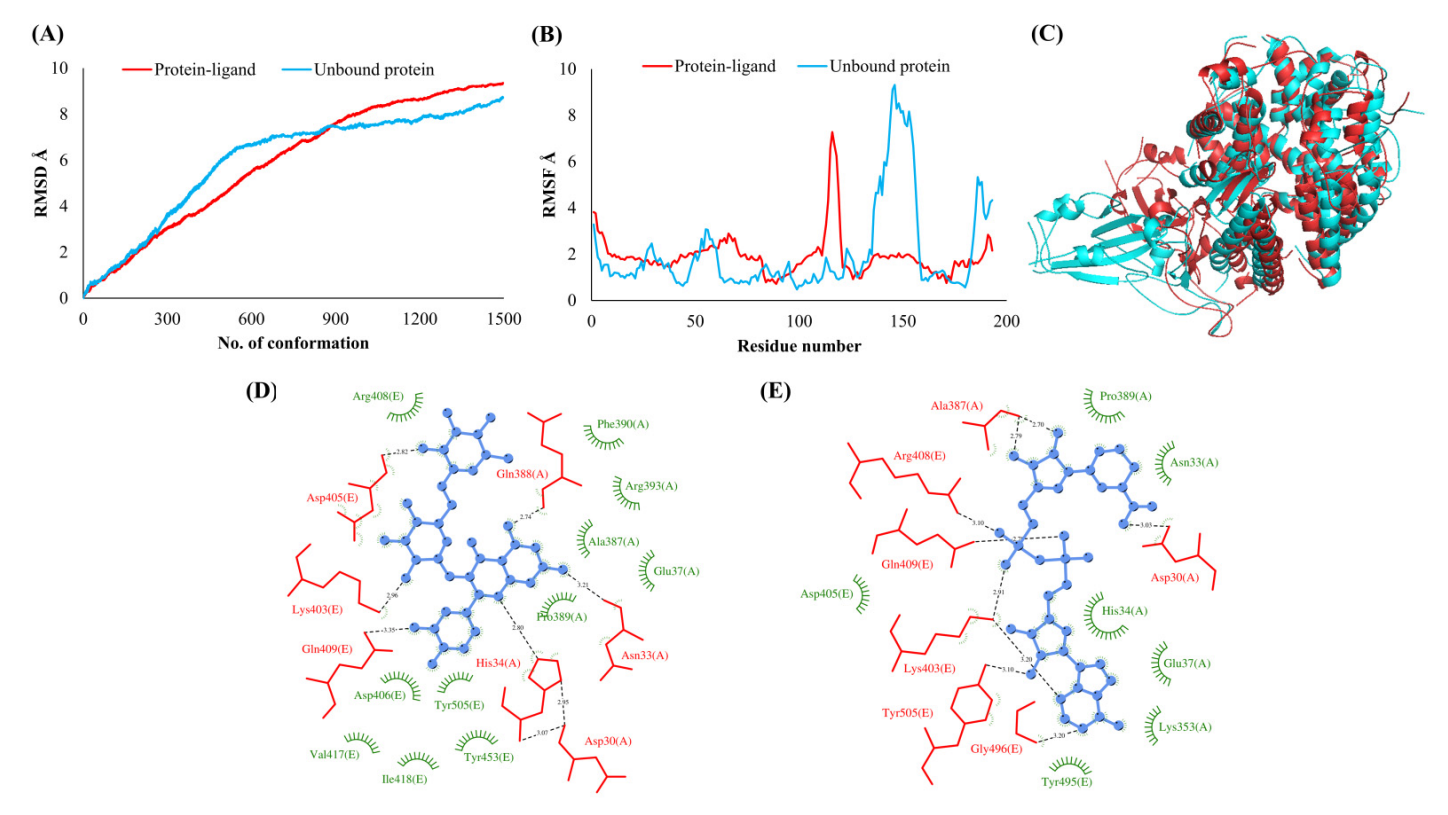
Spike (S). (**A**) RMSD plot of unbound and ligand-bound protein (**B**) RMSF plot of unbound and ligand-bound protein (**C**) Superimposition of unbound and ligand-bound protein (**D**) Molecular interactions of protein with Rutin (**E**) Molecular interactions of protein with NADH.

**Figure 10:**
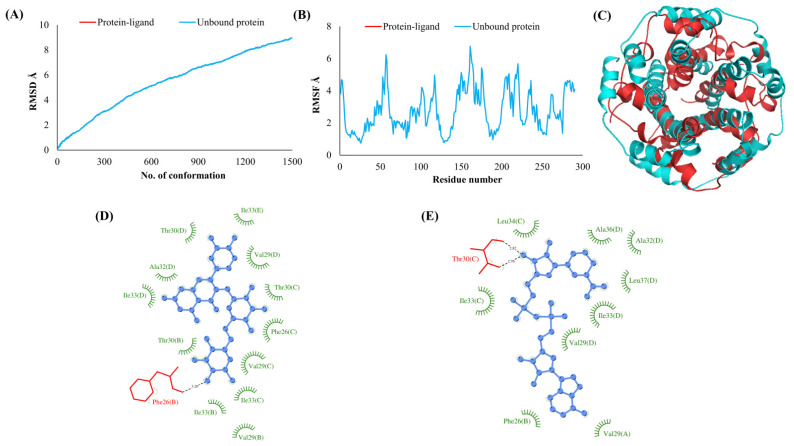
Envelope (E). (**A**) RMSD plot of unbound and ligand-bound protein (**B**) RMSF plot of unbound and ligand-bound protein (**C**) Superimposition of unbound and ligand-bound protein (**D**) Molecular interactions of protein with Rutin (**E**) Molecular interactions of protein with NADH.

**Figure 11:**
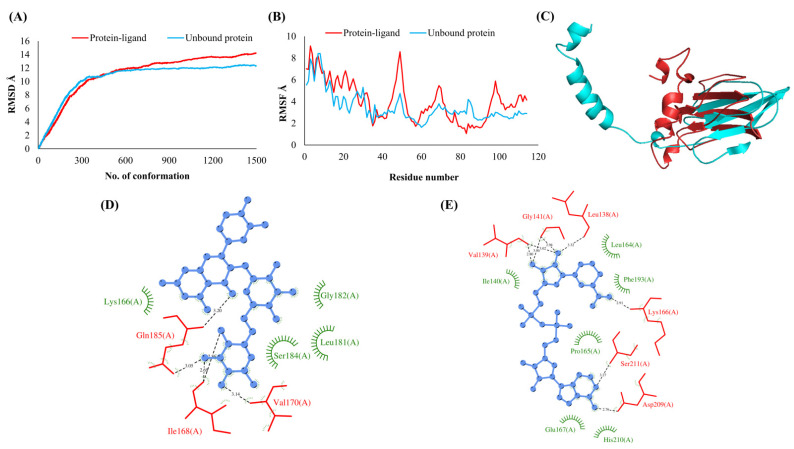
Membrane (M). (**A**) RMSD plot of unbound and ligand-bound protein (**B**) RMSF plot of unbound and ligand-bound protein (**C**) Superimposition of unbound and ligand-bound protein (**D**) Molecular interactions of protein with Rutin (**E**) Molecular interactions of protein with NADH.

**Figure 12:**
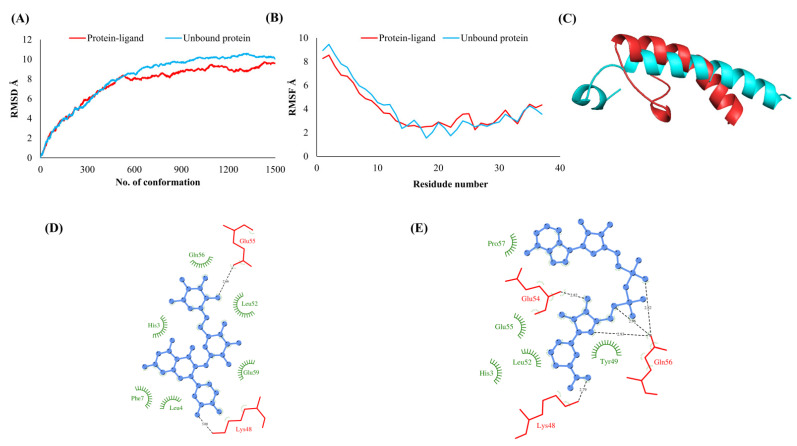
ORF6. (**A**) RMSD plot of unbound and ligand-bound protein (**B**) RMSF plot of unbound and ligand-bound protein (**C**) Superimposition of unbound and ligand-bound protein (**D**) Molecular interactions of protein with Rutin (**E**) Molecular interactions of protein with NADH.

**Figure 13:**
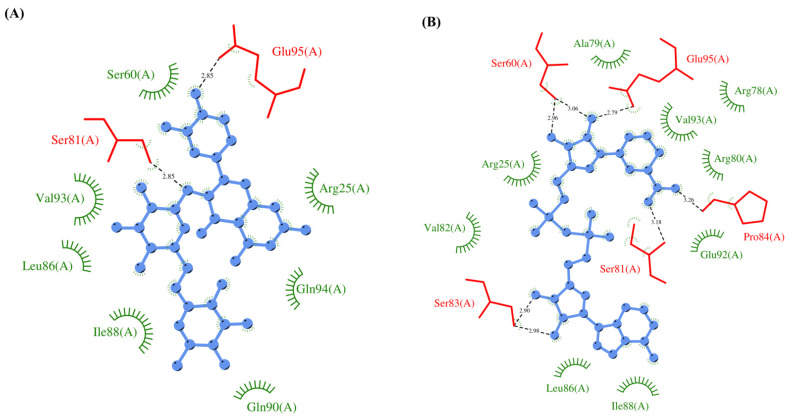
ORF7a. (**A**) Molecular interactions of protein with Rutin (**B**) Molecular interactions of protein with NADH.

**Figure 14:**
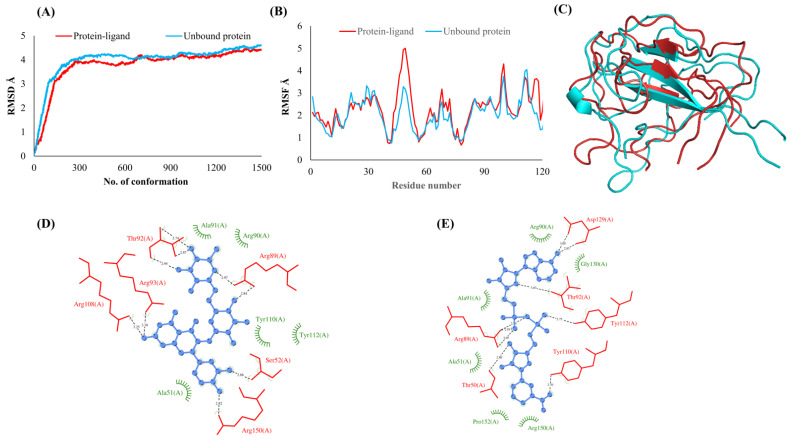
Nucleocapsid (N). (**A**) RMSD plot of unbound and ligand-bound protein (**B**) RMSF plot of unbound and ligand-bound protein (**C**) Superimposition of unbound and ligand-bound protein (**D**) Molecular interactions of protein with Rutin (**E)** Molecular interactions of protein with NADH.

The RMSDs of Cα atoms for proteins NSP1, NSP3, NSP5, NSP9, NSP12, NSP13, NSP15, ORF3a, S, E, M, ORF6, and N, in ligand-bound (closed) conformations vis-a-vis unbound (open in the absence of ligand) structures as a function of the number of conformations acquired by the proteins during the simulations, was computed after NMA (Figure 1). The graph clearly shows that large conformational transitions occurred at lower frequency (energy) modes in the case of all the proteins [19]. Among the 1500 conformations generated during each NMA run for every protein, the last 900–1500 confirmations represent the transition towards a closed structure. Upon comparison to ligand-free forms, as shown in Figure 1, Figure 2, Figure 3, Figure 4, Figure 5, Figure 6, Figure 7, Figure 8, Figure 9, Figure 10, Figure 11, Figure 12, Figure 13 and Figure 14, it is evident that the proteins (Figure 1A, Figure 3A, Figure 4A, Figure 8A, Figure 9A, Figure 10A, Figure 12A, Figure 13A and Figure 14A) except NSP3 (Figure 2A), NSP12 (Figure 5A), NSP13 (Figure 6A), NSP15 (Figure 7A) and M (Figure 11A), did not undergo large movements to attain closed conformations.

Similar results were observed in the case of RMSF graphs of Cα atoms for open and closed structures. The magnitude of residue wise fluctuations was lower in the case of protein-ligand complexes, NSP1 (Figure 1B), NSP3 (Figure 2B), NSP5 (Figure 3B), NSP15 (Figure 7B), ORF3a (Figure 8B), S (Figure 9B) and ORF6 (Figure 12B) in contrast to the proteins alone pointing towards the stabilizing effects of ligands. The residues in NSP5, NSP15, S, and ORF6 proteins showed distinctively less degree of fluctuations in the presence of drugs. Moreover, superimposition of the open and closed conformations of proteins also revealed noticeably compact and folded proteins in the presence of drugs (Figure 1, Figure 2, Figure 3, Figure 4, Figure 5, Figure 6, Figure 7, Figure 8, Figure 9, Figure 10, Figure 11, Figure 12, Figure 13 and Figure 14, panel C). An entire overlap in RMSD (Figure 10A) and RMSF (Figure 10B) data points for unbound- and ligand-bound protein was observed in the case of E protein. However, superimposition of open and closed structures showed signs of protein folding and compactness in the presence of ligand (Figure 10C).

### 3.3. Protein-Ligand Interaction Analyses

**Figure 15:**
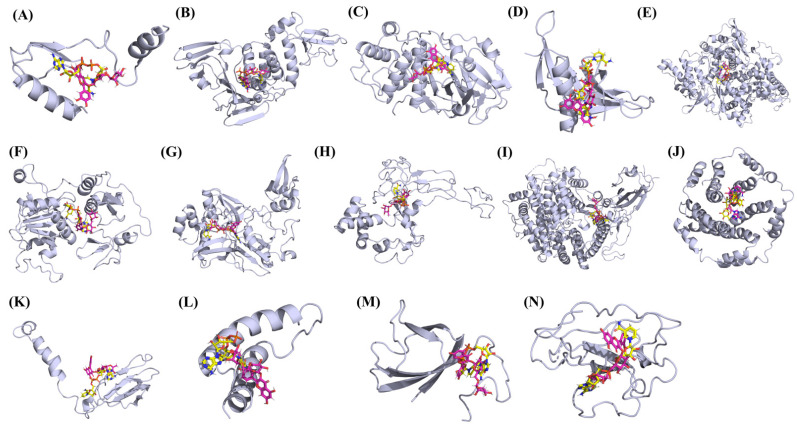
The binding mode of rutin (pink) and NADH (yellow) with the 14 different SARS-CoV-2 proteins. (**A**) NSP1. (**B**) NSP3. (**C**) NSP5. (**D**) NSP9. (**E**) NSP12. (**F**) NSP13. (**G**) NSP15. (**H**) ORF3a. (**I**) S. (**J**) E. (**K**) M. (**L**). ORF6. (**M**) ORF7a. (**N**) N Proteins.

**Figure 16:**
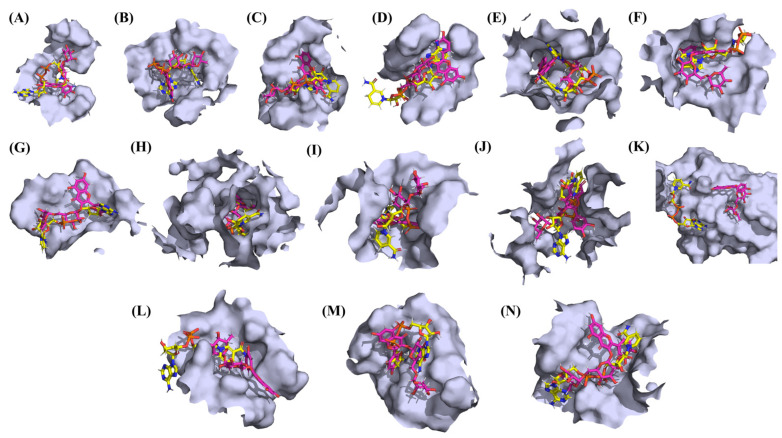
The binding of rutin (pink) and NADH (yellow) in the pockets of 14 different SARS-CoV-2 proteins. (**A**) NSP1. (**B**) NSP3. (**C**) NSP5. (**D**) NSP9. (**E**) NSP12. (**F**) NSP13. (**G**) NSP15. (**H**) ORF3a. (**I**) S. (**J**) E. (**K**) M. (**L**). ORF6. (**M**) ORF7a. (**N**) N Proteins.

Rutin formed six hydrogen bonds with NSP1 and NSP3, five with NSP5 and NSP9, twelve with NSP12, ten with NSP13, six with ORF3a, and nine with N protein. NADH majorly formed hydrogen bonds with NSP1 (seven), NSP3 (nine), NSP9 (twelve), NSP12 (eleven), NSP13 (thirteen), NSP15 (five), M (eight), ORF6 (five), and N protein (eight). Figure 1, Figure 2, Figure 3, Figure 4, Figure 5, Figure 6, Figure 7, Figure 8, Figure 9, Figure 10, Figure 11, Figure 12, Figure 13 and Figure 14, panels C and D illustrate the hydrogen bonds and hydrophobic interactions of NSP1, NSP3, NSP5, NSP9, NSP12, NSP13, NSP15, ORF3a, S, E, M, ORF6, ORF7a, and N proteins with Rutin and NADH, respectively. Figure 15 and Figure 16 illustrate the binding mode of Rutin and NADH with the 14 different SARS-CoV-2 proteins.

Several SARS-CoV-2 proteins with high mutational propensity have been identified, which further demands multi-targeted inhibitors [20]. Targeting multiple proteins with a single molecule would reduce the chances of resistance, and this approach will put forward an attractive model for drug development against COVID-19. Rutin, NADH, and Ginsenoside Rg1 showed high binding affinity against most of the SARS-CoV-2 proteins. Rutin is a flavonol glycoside found profusely in several plants and is a key component of nutritional supplements. Rutin has strong antioxidant properties and has been shown for its neuroprotective effect, anticarcinogenic and antidiabetic activities, treatment of cardiological and inflammatory diseases, and various other pharmacological activities [21,22]. NADH is a reduced coenzyme found extensively in nature and performs significant metabolic activities. NADH is being explored for its effectiveness in treating cardiovascular diseases, dementia-related Alzheimer’s and Parkinson’s disease, and chronic fatigue syndrome [23,24]. Several studies have mentioned Rutin as one of the potential inhibitors of SARS-CoV-2. Xu et al. 2020 showed the biding of Rutin with the main protease and common interaction sites, Asn142, Cys145, His164, Met165, Gln189, and Thr190 [25,26]. Another study reported interaction of Rutin with common interacting sites, Thr556, Tyr619, Lys621, Cys622, Asp623, Asn691, Asp761 of RNA-dependent RNA polymerase (NSP12); and Gly163, Arg166, Glu167, Asn267, Tyr273, of papain-like proteinase (NSP3) (Rahman et al., 2021). NADH has also been shown to be a possible inhibitor of the main protease and spike proteins of SARS-CoV-2 [27,28].

Ginsenoside Rg1 belongs to the ginsenosides class of compounds found in ginseng plants and has been used in traditional medicine for a long [29]. Ginsenoside Rg1 has been known to effect blood, cardiovascular, nervous, and immune systems performing various biological activities. The drug is also under clinical trials to treat dementia and cognitive impairments, rheumatic disorders, and stroke [30]. A large number of hydrogen bonding interactions and a robust hydrophobic network formed by these drugs with the proteins suggest their inhibitory effect on SARS-CoV-2. The candidate compounds proposed in the present study can be verified for inhibitory activity against SARS-CoV-2 and blocking viral-host interactions. This multi-targeted drug design approach will aid in global efforts to fight this pandemic by expediting drug development against COVID-19.

## 4. Conclusions

Studies conducted earlier in the development of SARS-CoV-2 have identified several proteins with a high mutational propensity, making multi-targeting inhibitors desirable. The present study involved the use of nutraceuticals against 14 different SARS-CoV-2 proteins, namely NSP1, NSP3, NSP5, NSP9, NSP12, NSP13, NSP15, ORF3a, S, E, M, ORF6, ORF7a, and N. Both virtual screening and model-based simulations have proved valuable in the identification of compounds that could be considered for the treatment of SARS-CoV-2. Consequently, hydrophobic networks and hydrogen bonds were observed between the protein and ligand as a consequence of the interaction analyses. The findings of this study will likely expedite the development of anti-COVID-19 drugs, which will contribute to global efforts to prevent the pandemic. 

## Figures and Tables

**Table 1:** Activity of drugs against SARS-CoV-2 proteins.

Drug	Description	Activity against ORFs
Rutin	An existing USFDA-approved drug used for strengthening weakened capillaries. Additionally, it has a powerful antioxidant with potential biological effect in reducing post-thrombotic syndrome, veins insufficiency or endothelial dysfunction.	NSP1, NSP3, NSP5, NSP9, NSP12, NSP13, NSP15, ORF3a, Spike, Envelope, Membrane, ORF6, ORF7a, Nucleocapsid
NADH	NADH plays essential metabolic roles and has been used to combat chronic fatigue syndrome. It is also being explored to be used against dementia and improving mental health.	NSP1, NSP3, NSP5, NSP9, NSP12, NSP13, NSP15, ORF3a, Spike, Envelope, Membrane, ORF6, ORF7a, Nucleocapsid
Ginsenoside Rg1, protopanaxatriol	Ginsenoside is a major component of the root and stem of ginseng plant. It possesses a broad spectrum of pharmacological properties such as neuroprotection, anti-inflammation, anti-aging, anti-fatigue and memory-enhancing properties.	NSP5, Nucleocapsid, NSP1, Envelope, NSP12, ORF5, ORF7a, NSP3, NSP9, NSP15

**Table 2:** Virtual screening results of nutraceuticals against SARS-CoV-2 proteins.

	Protein	Drug Name	Glide Score (kcal/mol)	GOLD Score	AutoDock Score (kcal/mol)
1	Spike (S)	NADH	−11.31	80.76	−8.4
		Rutin	−9.94	95.52	−6.7
2	Main protease (NSP5)	Ginsenoside Rb1	−9.37	132.14	−19.9
		Rutin	−9.58	96.35	−7.4
		NADH	−8.65	85.20	−8.5
		Ginsenoside Rg1	−8.16	110.03	−10.7
3	Nucleocapsid (N)	Rutin	−9.34	87.13	−5.6
		Ginsenoside C	−8.82	122.35	−12.0
		NADH	−8.13	70.55	−8.6
		Ginsenoside Rg1	−5.17	110.79	−10.7
4	ORF6	Ginsenoside C	−7.51	109.10	−13.5
		Spermine	−7.21	43.27	−6.8
		Rutin	−6.59	87.57	−5.7
		NADH	−4.98	60.11	−8.6
5	Leader protein (NSP1)	Ginsenoside C	−9.30	107.85	−11.6
		NADH	−7.38	65.42	−7.7
		Rutin	−7.09	80.41	−4.7
		Ginsenoside Rg1	−5.81	85.13	−9.4
6	Envelope (E)	Ginsenoside Rg1	−8.30	94.13	−11.5
		α−tocopherol succinate	−8.11	39.97	−16.7
		Rutin	−3.86	94.13	−11.5
		NADH	−3.08	68.11	−9.5
7	RNA−dependent RNA polymerase (NSP12)	Ginsenoside Rb1	−11.00	123.74	−19.6
		Rutin	−10.98	113.81	−7.0
		Ginsenoside Rg1	−10.14	143.41	−12.2
		NADH	−9.00	77.89	−9.5
8	ORF 3a	Rutin	−11.47	93.67	−7.5
		Ornithine	−9.10	39.31	−6.3
		NADH	−3.34	91.53	−10.0
9	Membrane (M)	Ginsenoside Rg1	−9.35	81.77	−9.4
		Rutin	−7.62	77.90	−5.5
		NADH	−5.40	58.68	−8.0
10	ORF 7a	Ginsenoside Rb1	−8.70	166.98	−16.8
		Ginsenoside Rg1	−7.00	135.96	−10.7
		NADH	−6.83	78.90	−7.6
		Rutin	−4.96	126.00	−5.0
11	Papain-like protease (NSP3)	Ginsenoside Rg1	−10.37	103.78	−10.2
		Rutin	−8.22	91.30	−6.7
		NADH	−6.63	80.28	−8.0
12	Helicase (NSP13)	Glutathione	−10.31	60.00	−6.4
		NADH	−9.54	87.21	−10.5
		Rutin	−7.85	85.62	−7.1
13	RNA binding protein (Orf1ab, nsp9)	Ginsenoside Rb1	−7.92	102.08	−20.1
		Ginsenoside Rg1	−6.84	91.46	−9.6
		Rutin	−6.56	70.25	−5.1
		NADH	−5.66	55.60	−7.9
14	Endoribonuclease (NSP15)	Ginsenoside Rb1	−13.50	110.76	−24.4
		Ginsenoside Rg1	−11.00	111.16	−12.5
		NADH	−9.95	78.94	−9.2
		Rutin	−9.60	100.05	−7.1

## Data Availability

All the data available is provided in this paper.
